# Anomalous Behavior Induced by a Single Impurity in Non-Hermitian Topological Systems with Nonreciprocal Coupling

**DOI:** 10.3390/e28050572

**Published:** 2026-05-19

**Authors:** Junjie Wang, Zhenyan Wang, Xie Ma, Xuexi Yi

**Affiliations:** 1 College of Mechanical Engineering, Ningbo University of Finance & Economics, Ningbo 315175, China; wangjj012@nenu.edu.cn (J.W.); emri@sina.com (Z.W.); 2 School of Artificial Intelligence, Ningbo University of Finance & Economics, Ningbo 315175, China; maxie88@163.com; 3 Center for Quantum Sciences and School of Physics, Northeast Normal University, Changchun 130024, China

**Keywords:** non-Hermitian topological system, impurity, nonreciprocal coupling, skin effect

## Abstract

A remarkable feature of non-Hermitian topological systems with skin effects is that their spectra and eigenstates are strongly dependent on the choice of boundary conditions. Here, we investigate a system where the impurity couples to a nonreciprocal Su–Schrieffer–Heeger (SSH) chain at two points with nonreciprocal coupling. We first study the spectrum of the system and demonstrate that nonreciprocal couplings between the impurity and the chain alter its spectral structure. Particularly, this effect becomes particularly prominent in the limit of unidirectional coupling, inducing a shift in the parameter regime for the zero mode. Meanwhile, the impurity–chain couplings give rise to two effective boundary conditions and determine the spatial distribution of the zero mode. In addition, the localization of bulk states is significantly altered by tuning the nonreciprocity of the impurity–chain coupling. Notably, in the unidirectional coupling regime, two distinct types of bulk states coexist near the same boundary, one differing from the other in both spatial distribution and degree of localization. We also find that the bulk states undergo significant skin phase transitions as the coupling strength varies, characterized by a transition from conventional skin states to bipolar skin states. Our findings establish the feasibility of controlling non-Hermitian topological systems by coupling an impurity.

## 1. Introduction

Non-Hermitian physics has garnered considerable attention over the past few decades, revealing a diverse array of phenomena and applications in both classical and quantum systems [1,2,3,4,5]. A central focus of research in recent years has been on topological phases in non-Hermitian systems [6,7,8,9,10,11,12], which led to the discovery of the breakdown of the conventional bulk-boundary correspondence [13,14]. Subsequent work settled this issue and introduced a generalized bulk-boundary correspondence [15,16]. Over the years, a broad range of theoretical and experimental investigations have been carried out on this topic in numerous physical settings [17,18,19,20,21,22,23,24,25,26,27,28,29,30,31,32,33,34,35,36,37].

Non-Hermitian topological systems exhibit a wealth of unique behaviors for which no counterparts can be found in Hermitian systems. One of these distinctive property is the localization of all eigenstates at the boundaries, which is a phenomenon termed the “non-Hermitian skin effect” [16]. This boundary effect results in the breakdown of the conventional bulk-boundary correspondence related to point-gap topology [17,18,19,20,21,22,23]. One notable characteristic of non-Hermitian systems with skin effects is that the behavior of both their spectra and eigenstates can be drastically altered by switching the boundary conditions from periodic to open. In between these limits, an impurity embedded in the system can also serve as an effective boundary. The influence of a single local impurity on physical properties has become a topic of significant interest in the field of non-Hermitian topology [38,39,40,41,42,43,44,45,46,47,48]. Ref. [38] first reported that nonreciprocal impurities embedded in a non-Hermitian Hatano–Nelson chain can induce scale-free localization. Furthermore, eigenstates were found to emerge either along or opposite to the direction of nonreciprocity, depending on the impurity strength regime. On-site impurities in non-Hermitian topological chains have also been considered in Ref. [41]. It is shown that increasing the impurity strength drives a transition of the bulk states from nonskin to skin states. Additionally, since the energy shift of the system can be dramatically changed by adding a tiny boundary impurity, this kind of system has even been proposed for exponentially enhanced quantum sensing in an experimentally realistic setting [49,50,51,52,53].

In circuit systems, the superconducting qubit can be coupled to the topological circuit at multiple sites [54,55,56], so this qubit is called the giant atom. On the other hand, the superconducting qubit can also be replaced by a single node in circuit systems. This motivates the introduction of a new type of impurity in non-Hermitian topological systems, dubbed the “coupling impurity” [57]. Unlike the first two types of impurities, including on-site impurities and nonreciprocal impurities, which are single and localized, the coupling impurity is characterized by its connection to the topological chain via multiple coupling points. This nonlocal coupling enables the formation of multiple effective boundary conditions. Additionally, the coupling configuration can be deliberately engineered to control the topological characteristics of systems. It has been found that non-Hermitian topological systems with coupling impurities can be applied to the design of multi-parameter sensors [58]. However, the couplings between the impurity and the chain are typically Hermitian. So, this begs the question: what new physics would emerge if the impurity–chain couplings are rendered non-Hermitian?

In this work, we focus on a system composed of a two-level impurity and a nonreciprocal SSH chain with nonreciprocal couplings. Interestingly, the nonreciprocal nature of the impurity–chain couplings drive a reshaping of the spectrum, ultimately leading to a shift in the parameter regime for the zero mode in the unidirectional coupling limit. We also find that the nonreciprocity of the impurity–chain couplings determine whether the zero mode localizes at one effective boundary or both of them. Further examination shows that impurity–chain couplings can induce skin phase transitions from conventional skin states to bipolar skin states. Another unprecedented finding is that two distinct types of localized bulk states emerge at the same effective boundary in the unidirectional coupling regime.

## 2. Model

We consider a nonreciprocal SSH chain with periodic boundary conditions (PBC). The Hamiltonian of this system can be written as

(1) $$\begin{array}{c} H_{\mathrm{SSH}}=\sum_{l=1}^{L}\left[\left(t_{1}+\gamma \right){\hat{C}}_{A,l}^{†}{\hat{C}}_{B,l}+\left(t_{1}-\gamma \right){\hat{C}}_{B,l}^{†}{\hat{C}}_{A,l}+t_{2}{\hat{C}}_{A,l+1}^{†}{\hat{C}}_{B,l}+t_{2}{\hat{C}}_{B,l}^{†}{\hat{C}}_{A,l+1}\right], \end{array}$$

where ${\hat{C}}_{A(B),l}^{†}$ and ${\hat{C}}_{A(B),l}$ are the creation and annihilation operators for the sublattice site A(B) at *l*-th unit cell. *L* is the overall number of unit cells. $t_{1}\pm \gamma$ and $t_{2}$ are staggered nearest-neighbor hopping amplitudes. The asymmetry of hopping amplitudes (γ≠0) leads to the non-Hermiticity of the system.

**Figure 1:**
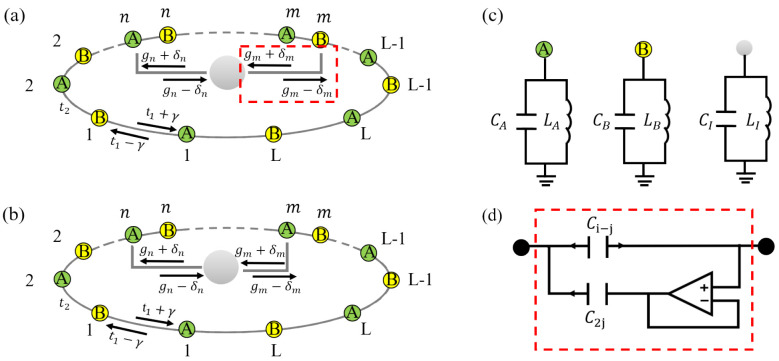
Sketch of the system. (**a**) A single impurity coupled to a nonreciprocal SSH chain via A−B couplings. (**b**) A single impurity coupled to a nonreciprocal SSH chain via A−A couplings. (**c**) In a circuit system, the internal structure of groundings for the three types of nodes, including A node, B node, and impurity node. These correspond to the A site, B site, and the impurity site, respectively, in (**a**) and (**b**). (**d**) In a circuit system, the internal structure of nonreciprocal capacitance on two nodes.

To control the aforementioned non-Hermitian topological system, we introduce an impurity coupled to two sites (*n* and *m*) of a nonreciprocal SSH chain via A−B couplings [Figure 1a] or A−A couplings [Figure 1b]. Without loss of generality, we hereafter assume n<m. A notable feature is that the impurity–chain coupling is also nonreciprocal. The interaction Hamiltonian of the impurity–chain coupling is described as follows

(2) $$\begin{array}{cc} H_{I,AB} & =\left(g_{n}-\delta_{n}\right)\sigma^{+}{\hat{C}}_{A,n}+\left(g_{n}+\delta_{n}\right)\sigma^{-}{\hat{C}}_{A,n}^{†}+\left(g_{m}+\delta_{m}\right)\sigma^{+}{\hat{C}}_{B,m}+\left(g_{m}-\delta_{m}\right)\sigma^{-}{\hat{C}}_{B,m}^{†},\\ H_{I,AA} & =\left(g_{n}-\delta_{n}\right)\sigma^{+}{\hat{C}}_{A,n}+\left(g_{n}+\delta_{n}\right)\sigma^{-}{\hat{C}}_{A,n}^{†}+\left(g_{m}+\delta_{m}\right)\sigma^{+}{\hat{C}}_{A,m}+\left(g_{m}-\delta_{m}\right)\sigma^{-}{\hat{C}}_{A,m}^{†}, \end{array}$$

where $g_{n}\pm \delta_{n}$ ($g_{m}\pm \delta_{m}$) denote nonreciprocal coupling strengths between the impurity and SSH chain. $\sigma^{+}=\left|e\rangle \langle g\right|$ is the usual pseudospin ladder operator. |g〉 and |e〉 are the ground state and the excited state of the impurity, respectively. The total Hamiltonians of the system as schematically shown in Figure 1 can be expressed as

(3) $$H_{AB}=H_{\mathrm{SSH}}+H_{I,AB},$$

(4) $$H_{AA}=H_{\mathrm{SSH}}+H_{I,AA}.$$

Here, we have assumed that the impurity is resonant with the energy band center, i.e., frequency of impurity is zero. For the experimental scheme, adding a constant imaginary shift to all sites corresponding to a passive setting with loss only [59], this correction does not affect the localization of eigenstates and existence of boundary modes. This system can be physically realized in electrical circuits. The non-Hermitian topological circuit employs 2L+1 nodes, including *L* A nodes, *L* B node, and a single impurity node. In Figure 1c, we show the internal structure for the three types of nodes, which are composed of grounded LC circuits. As shown in Figure 1d, nonreciprocal couplings between the impurity and the chain are engineered by placing an effective nonreciprocal capacitance ($C_{i}\pm C_{j}$). The nonreciprocal capacitor is composed of two parts. One is realized by connecting a capacitor ($2C_{j}$) in series with a voltage follower, which is constructed from an operational amplifier and a negative feedback network, the other is a capacitor ($C_{i}-C_{j}$) in parallel [33,52]. Due to the virtual open and virtual short circuit conditions between the inverting input and noninverting input pins, the current at the one side of the capacitor $2C_{j}$ is blocked, while it remains uninfluenced at the other side. In addition, the intercell coupling of the circuit is fulfilled by a capacitor, and the intracell coupling is also achieved by an effective nonreciprocal capacitor as shown in Figure 1d. The capacitance can vary over a broad range (e.g., from pF to nF), making the transition of nonreciprocal coupling readily achievable in the experiment.

## 3. Results

### 3.1. Spectrum

**Figure 2:**
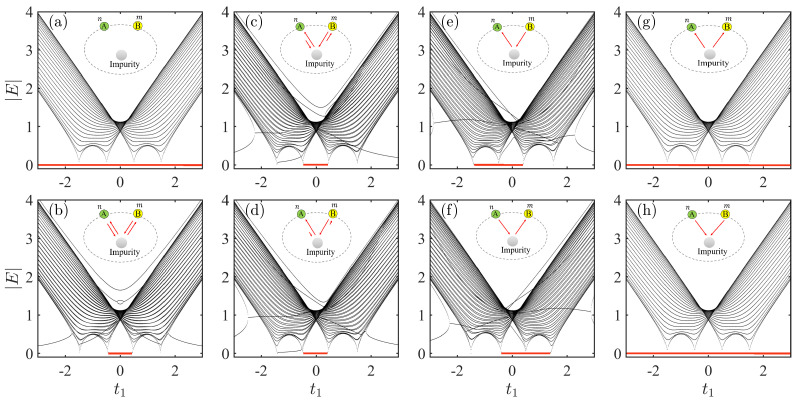
Absolute value of the spectra for the system via A−B couplings as a function of $t_{1}$ under different impurity–chain coupling strengths. (**a**) The spectrum under decoupling limits with $g_{n}=g_{m}=0$, and $\delta_{n}=\delta_{m}=0$. (**b**) The spectrum under equal coupling strengths with $g_{n}=g_{m}=1$, and $\delta_{n}=\delta_{m}=0$. (**c**,**d**) The spectra under nonreciprocal couplings with $g_{n}=g_{m}=1$. Parameters: $\delta_{n}=\delta_{m}=0.5$ (**c**); $\delta_{n}=\delta_{m}=0.7$ (**d**). (**e**–**h**) The spectra under four different unidirectional coupling regimes with $g_{n}=g_{m}=1$. Parameters: $\delta_{n}=\delta_{m}=1$ (**e**); $\delta_{n}=\delta_{m}=-1$ (**f**); $\delta_{n}=-\delta_{m}=1$ (**g**); $\delta_{n}=-\delta_{m}=-1$ (**h**). For each case, a corresponding sketch of the system is inserted, where the red arrows indicate the direction of coupling. The results are obtained by numerically solve the Schrödinger equation. The parameters shared by all the figures are taken as $L=50,n=25,m=26,t_{1}=0.2,t_{2}=1,$ and γ=0.5.

Our analysis begins with the system (3) where an impurity is coupled to a nonreciprocal SSH chain via A−B couplings. To illustrate the role of nonreciprocal coupling between the impurity and SSH chain, we show the absolute value of the spectrum for the system as a function of $t_{1}$ under different coupling strengths in Figure 2.

For comparison, we first consider the decoupling limit by setting $g_{n}=g_{m}=\delta_{n}=\delta_{m}=0$, with the corresponding spectra presented in Figure 2a. In this scenario, impurity and nonreciprocal SSH chain are separable. Note that there is an isolated zero energy (|E|=0), which remains unchanged with the parameter $t_{1}$. As the nonreciprocal SSH chain under PBC lacks zero energy, the zero energy must originate from the isolated impurity. Furthermore, the other parts in Figure 2a correspond exactly to the spectrum of the original nonreciprocal SSH chain under PBC. In Figure 2b, we show the spectrum of the system under equal coupling strengths with $g_{n}=g_{m}=1$, and $\delta_{n}=\delta_{m}=0$. In contrast to Figure 2a, a distinct feature here is the emergence of a topologically protected zero energy. In the long-chain limits, this mode exists when the parameter $t_{1}$ falls within the interval $t_{1}\in \left[-t_{2}+\gamma ,t_{2}-\gamma \right]$ [56]. This suggests that the impurity serves as an effective boundary for the nonreciprocal SSH chain.

We now proceed to investigate the spectrum of systems through controlling the nonreciprocity of the impurity–chain coupling. In Figure 2c, we show the spectrum of systems under nonreciprocal couplings with parameters $g_{n}=g_{m}=1$, and $\delta_{n}=\delta_{m}=0.5$. It can be seen that the condition for the emergence of the zero energy coincides with the criterion previously established in the case of equal coupling strengths. However, this criterion breaks down upon gradually enhancing the nonreciprocity of the coupling, as shown in Figure 2c–e. In particular, as shown in Figure 2e, a key feature is that the parameter range for the emergence of the zero energy is modified in the unidirectional coupling regime with $g_{n}=g_{m}=1$, and $\delta_{n}=\delta_{m}=1$. Additionally, a further modification occurs in another unidirectional coupling regime with $\delta_{n}=\delta_{m}=-1$, as shown in Figure 2f. This demonstrates that unidirectional coupling can induce a shift in the parameter regime for the zero mode.

The aforementioned scenarios for unidirectional couplings both represent cases of couplings in the same direction ($\delta_{n}$ and $\delta_{m}$ share the same sign). In additions, as shown in Figure 2g,h, there is always an isolated zero energy for the system with unidirectional couplings in the opposite direction as setting $\delta_{n}=-\delta_{m}=1$, or $\delta_{n}=-\delta_{m}=-1$. The most counterintuitive finding is that the spectrum in Figure 2g,h matches the decoupled scenario exactly as shown in Figure 2a.

To understand above anomalous behaviors and give the parameter range of the zero energy in the long chain limits, we present the following analysis, based on algebraic calculations. By applying the Fourier transform to Equation (1), the Hamiltonian of a nonreciprocal SSH chain under PBC in the momentum space reads

(5) $$\begin{array}{cc} H_{\mathrm{SSH}}\left(k\right)= & \sum_{k}\left[\left[\left(t_{1}+\gamma \right)+t_{2}e^{-ik}\right]{\hat{C}}_{A,k}^{†}{\hat{C}}_{B,k}+\left[\left(t_{1}-\gamma \right)+t_{2}e^{ik}\right]{\hat{C}}_{B,k}^{†}{\hat{C}}_{A,k}\right]. \end{array}$$

The corresponding spectrum can be given as

(6) $$\begin{array}{c} E\left(k\right)=\pm \sqrt{\left[\left(t_{1}+\gamma \right)+t_{2}e^{-ik}\right]\left[\left(t_{1}-\gamma \right)+t_{2}e^{ik}\right]}\equiv \pm \omega_{k}. \end{array}$$

Then, the total Hamiltonian of the system (3) via A−B couplings in the momentum space can be described as

(7) $$\begin{array}{c} H_{AB}\left(k\right)=H_{\mathrm{SSH}}\left(k\right)+H_{I,AB}\left(k\right), \end{array}$$

with

(8) $$\begin{array}{cc} H_{I,AB}\left(k\right)=\frac{1}{\sqrt{L}}\sum_{k} & [\left(g_{n}-\delta_{n}\right)e^{ikn}\sigma^{+}{\hat{C}}_{A,k}+\left(g_{n}+\delta_{n}\right)e^{-ikn}\sigma^{-}{\hat{C}}_{A,k}^{†}\\ \; & +\left(g_{m}+\delta_{m}\right)e^{ikm}\sigma^{+}{\hat{C}}_{B,k}+\left(g_{m}-\delta_{m}\right)e^{-ikm}\sigma^{-}{\hat{C}}_{B,k}^{†}]. \end{array}$$

Making use of the time-independent Schrödinger equation $H_{AB}\left(k\right)|\psi \rangle =E\left(k\right)|\psi \rangle$, we obtain the transcendental equation for the energy E(k) of the system, which can be written as (See Appendix A for analytical results)

(9) $$\begin{array}{cc} E=\frac{1}{L}\sum_{k} & \frac{\left(g_{n}-\delta_{n}\right)e^{ikn}}{E^{2}-\omega_{k}^{2}}\left[\left(g_{n}+\delta_{n}\right)e^{-ikn}E+\left(g_{m}-\delta_{m}\right)e^{-ikm}\left(t_{1}+\gamma +t_{2}e^{-ik}\right)\right]\\ +\frac{1}{L}\sum_{k} & \frac{\left(g_{m}+\delta_{m}\right)e^{ikm}}{E^{2}-\omega_{k}^{2}}\left[\left(g_{m}-\delta_{m}\right)e^{-ikm}E+\left(g_{n}+\delta_{n}\right)e^{-ikn}\left(t_{1}-\gamma +t_{2}e^{ik}\right)\right]. \end{array}$$

It is straightforward to see that substituting the couplings defined by $g_{n}=\pm \delta_{n}$ and $g_{m}=\mp \delta_{m}$ into Equation (9) leads to a simplified form:

(10) $$\begin{array}{cc} \sum_{k}E\left(E^{2}-\omega_{k}^{2}\right) & =0, \end{array}$$

This immediately yields the energy solutions E=0 or $E=\pm \omega_{k}$. Note that $E=\pm \omega_{k}$ corresponds to the spectrum of a nonreciprocal SSH chain under PBC, given in Equation (6). This suggests that unidirectional couplings in the opposite direction give rise to an isolated zero energy and has no impact on the overall bulk band structure. This confirms that the spectra in Figure 2g,h and Figure 2a are identical.

**Figure 3:**
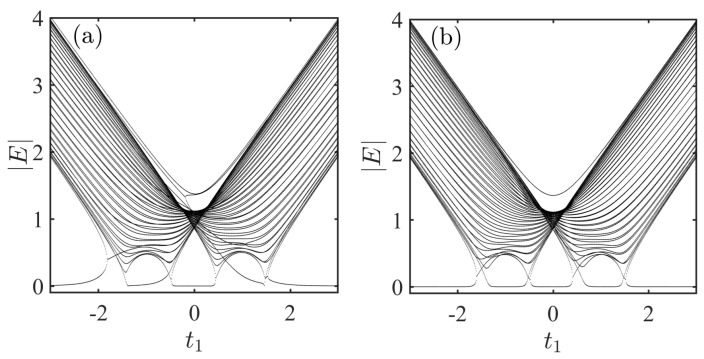
Absolute value of the spectra for the system via A−B couplings as a function of $t_{1}$ under different impurity–chain coupling sites. (**a**) The spectrum with n=25, m=30. (**b**) The spectrum with n=1, m=25. The results are obtained by numerically solve the Schrödinger equation. The parameters shared by all the figures are taken as $L=50,g_{n}=1,g_{m}=1,\delta_{n}=0.5,\delta_{m}=0.5$, $t_{1}=0.2,t_{2}=1,$ and γ=0.5.

In Figure 3, we show the absolute value of the spectra for the system via A−B couplings as a function of $t_{1}$ under different impurity–chain coupling sites. The impurity–chain coupling sites are taken as n=25, m=30 in Figure 3a, and n=1, m=25 in Figure 3b. Notably, an isolated energy level converges toward zero as the separation between the two coupling sites increases. Physically, the system can be regarded as two open chains decoupled by the impurity. Their combined spectrum dictates the behavior of systems. As m−n→+∞, the distinct A−B and B−A open boundaries inevitably induce a zero energy under arbitrary parameters [55].

We next derive the solution for the emergence of the zero energy in a generalizable case. To this end, we set E=0 in Equation (9), then both sides of this equation can be simplified via the residue theorem as follows (See Appendix A for analytical results)

(11) $$\begin{array}{cc} 0= & \left\{\begin{array}{cc} \frac{\alpha }{t_{2}}{\left(\frac{-t_{2}}{t_{1}-\gamma }\right)}^{m-n+1}, & (-t_{2}-\gamma <t_{1}<-t_{2}+\gamma ),\\ 0, & (-t_{2}+\gamma <t_{1}<t_{2}-\gamma ),\\ \frac{\beta }{t_{2}}{\left(\frac{-t_{2}}{t_{1}+\gamma }\right)}^{m-n+1}, & (t_{2}-\gamma <t_{1}<t_{2}+\gamma ),\\ \frac{\alpha }{t_{2}}{\left(\frac{-t_{2}}{t_{1}-\gamma }\right)}^{m-n+1}+\frac{\beta }{t_{2}}{\left(\frac{-t_{2}}{t_{1}+\gamma }\right)}^{m-n+1}, & (t_{1}<-t_{2}-\gamma \;or\;t_{1}>t_{2}+\gamma ). \end{array}\right. \end{array}$$

Here, $\alpha =\left(g_{m}-\delta_{m}\right)\left(g_{n}-\delta_{n}\right)$, $\beta =\left(g_{m}+\delta_{m}\right)\left(g_{n}+\delta_{n}\right)$, and it is assumed that $t_{2}>\gamma >0$. Equation (11) demonstrates that E=0 is the solution of Equation (9) only for $-t_{2}+\gamma <t_{1}<t_{2}-\gamma$. This is the parameter range for the emergence of the zero energy in the nonreciprocal coupling case as shown in Figure 2c,d. However, this parameter range is modified upon taking the unidirectional coupling between the impurity and the chain.

Here, the condition for the emergence of the zero energy and its range of parameters are presented as shown in the Table 1. One can clearly identify the parameter range where zero mode appear in each of the four different conditions. The first row represents the nonreciprocal coupling scenario discussed previously. In scenario II, the only requirement is that parameter α vanishes, i.e., $\left(g_{m}-\delta_{m}\right)\left(g_{n}-\delta_{n}\right)=0$. This condition is equivalent to satisfying either $g_{m}-\delta_{m}=0$ or $g_{n}-\delta_{n}=0$. A striking finding is that it is not necessary to satisfy unidirectional coupling, simultaneously, as illustrated in the Figure 2e. In a broader context, the shift in the parameter regime for the zero mode occurs as long as at least one of the two couplings is unidirectional. Similarly, scenario III follows an analogous analysis, so we omit the detailed discussion here. In scenario IV, it is necessary that both criteria α=0 and β=0 are met. The third column in Table 1 gives the corresponding numerical simulation results as presented earlier. This result establishes nonreciprocal impurity–chain coupling as a mechanism for inducing the shift in the parameter regime for the zero mode.

When γ=0, the system reduces to the Hermitian limit [55]. The topological property of the system is characterized by the winding number $w=\frac{1}{\pi }\int_{-\pi }^{\pi }dk\langle \phi \left|i\partial_{k}\right|\phi \rangle$. In this case, w=1 for $t_{1}<t_{2}$, w=0 for $t_{1}>t_{2}$. The parameter ranges for the emergence of the zero mode is identical to the non-trivial phase boundary w=1. Furthermore, the parameter range of zero modes in the proposed non-Hermitian impurity system is related to the non-Hermitian winding number. In the absence of the impurity, the different topological phases for a pure nonreciprocal SSH model can be distinguished by the non-Hermitian winding number $v=\frac{1}{\pi }\int_{-\pi }^{\pi }dk\langle \phi^{L}\left|i\partial_{k}\right|\phi^{R}\rangle$, where $\langle \phi^{L}|$ and $|\phi^{R}\rangle$ are the left and the right eigenstates of a pure nonreciprocal SSH model. Assuming $t_{2}>\gamma >0$, it satisfies

(12) $$v=\left\{\begin{array}{cc} 1, & (-t_{2}+\gamma <t_{1}<t_{2}-\gamma ),\\ \frac{1}{2}, & \left(-t_{2}-\gamma <t_{1}<-t_{2}+\gamma \right)\\ \; & or\;(t_{2}-\gamma <t_{1}<t_{2}+\gamma ),\\ 0, & \left(t_{1}<-t_{2}-\gamma \right)\;or\;\left(t_{1}>t_{2}+\gamma \right). \end{array}\right.$$

The corresponding values of the winding number have been inserted into Table 1. The Table 1 presents the parameter ranges of the zero modes under four different conditions. In scenario I, the parameter ranges for the emergence of the zero mode [$t_{1}\in \left(-t_{2}+\gamma ,t_{2}-\gamma \right)$] is identical to the non-trivial phase boundary of a pure SSH chain without the impurity v=1. However, in scenarios II–IV, there is no clear correspondence. In scenario II–III, the corresponding values of the winding number are 1/2, 1. Additionally, in scenario IV, the corresponding values of the winding number are 0, 1/2, 1.

Our focus now shifts to the study of the system (4), where an impurity is coupled to a nonreciprocal SSH chain via A−A couplings. The corresponding spectra in the momentum space satisfies (See Appendix A for analytical results)

(13) $$\begin{array}{cc} E= & \sum_{k}\frac{E[\left(g_{n}+\delta_{n}\right)\left(g_{m}+\delta_{m}\right)e^{ik(m-n)}]}{L(E^{2}-\omega_{k}^{2})}+\sum_{k}\frac{E[\left(g_{n}-\delta_{n}\right)\left(g_{m}-\delta_{m}\right)e^{ik(n-m)}]}{L(E^{2}-\omega_{k}^{2})}\\ + & \sum_{k}\frac{E[g_{n}^{2}+g_{m}^{2}-\delta_{n}^{2}-\delta_{m}^{2}]}{L(E^{2}-\omega_{k}^{2})}. \end{array}$$

**Figure 4:**
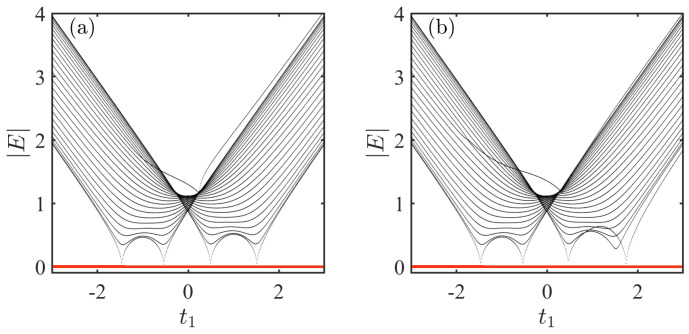
Absolute value of the spectra for the system via A−A couplings as a function of $t_{1}$ under different coupling strengths. Nonreciprocal coupling parameters are set as $\delta_{n}=\delta_{m}=0.5$ in (**a**) and $\delta_{n}=\delta_{m}=1$ in (**b**). The results are obtained by numerically solve the Schrödinger equation. The parameters shared by all the figures are taken as $L=50,n=25,m=26,g_{n}=1,g_{m}=1$, $t_{1}=0.2,t_{2}=1,$ and γ=0.5.

Obviously, E=0 is always the solution of Equation (13). This implies the existence of a robust zero energy in the system, unaffected by parameter variations. The numerical spectra for the system as a function of $t_{1}$ under different coupling strengths are shown in Figure 4a,b. Fortunately, this system still respects chiral symmetry $\Gamma^{-1}H_{AA}\Gamma =-H_{AA}$ with $\Gamma =\mathrm{diag}{\left(1,-1,1,-1,...,1,-1,-1\right)}_{2L+1}$, which causes the energies appear in the paired form of (E,−E). Combined with the condition that the system dimension is odd, this ensures the existence of a zero energy forever.

### 3.2. Spatial Transformation of Zero Mode

In this section, we investigate the spatial transformation of zero mode by tuning the couplings between the impurity and nonreciprocal SSH chain. We first consider the case of A−B coupling, followed by a brief discussion of the A−A coupling configuration.

**Figure 5:**
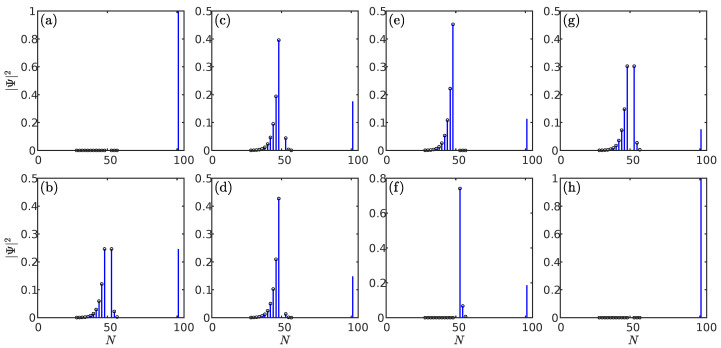
Spatial profile of zero mode for the system via A−B coupling as a function of site *N*. (**a**) Zero mode under decoupling limits with $g_{n}=g_{m}=0$, and $\delta_{n}=\delta_{m}=0$. (**b**) Zero mode under equal coupling strengths with $g_{n}=g_{m}=1$, and $\delta_{n}=\delta_{m}=0$. (**c**,**d**) Zero mode under nonreciprocal couplings with $g_{n}=g_{m}=1$. Parameters: $\delta_{n}=\delta_{m}=0.5$ (**c**); $\delta_{n}=\delta_{m}=0.7$ (**d**). (**e**–**h**) Zero mode under four different unidirectional coupling regimes with $g_{n}=g_{m}=1$. Parameters: $\delta_{n}=\delta_{m}=1$ (**e**); $\delta_{n}=\delta_{m}=-1$ (**f**); $\delta_{n}=-\delta_{m}=1$ (**g**); $\delta_{n}=-\delta_{m}=-1$ (**h**). The results are obtained by numerically solve the Schrödinger equation. The parameters shared by all figures are taken as $L=50,n=25,m=26,t_{1}=0.2,t_{2}=1,$ and γ=0.5. The site of impurity is set to N=2L+1=101.

To this end, the spatial profile of zero modes for the system via A−B coupling as a function of site *N* with different impurity–chain couplings are shown in Figure 5a–h. The site of impurity is set to N=2L+1=101, and the parameters are the same as used in Figure 2a–h. The bars represent the numerical results, and the empty circles represent the analytical results. Simple algebra shows that the analytical results of the probability amplitudes of zero modes satisfy (see Appendix B for details)

(14) $$\begin{array}{cc} \; & \frac{A_{l}}{U_{e}}=\left\{\begin{array}{cc} \frac{g_{m}-\delta_{m}}{t_{1}-\gamma }{\left(-\frac{t_{1}-\gamma }{t_{2}}\right)}^{\left(l-m\right)}, & (l>m),\\ 0, & (l\leq m), \end{array}\right.\\ \; & \frac{B_{l}}{U_{e}}=\left\{\begin{array}{cc} \frac{g_{n}+\delta_{n}}{t_{1}+\gamma }{\left(-\frac{t_{1}+\gamma }{t_{2}}\right)}^{\left(n-l\right)}, & (l<n),\\ 0, & (l\geq n), \end{array}\right. \end{array}$$

where $A_{l}\left(B_{l}\right)$ denotes the amplitude in sublattice site A(B) of the *l*th unit cell, and $U_{e}$ denotes the amplitude in site of the impurity. In Figure 5, it is clear that the analytical results (empty circles) given by Equation (14) are in good agreement with the numerical results (bars). Notably, one can manipulate the spatial profile of the zero mode by tuning the nonreciprocal couplings between the impurity and the chain.

As a comparison, we first consider the decoupling limit by setting $g_{n}=g_{m}=\delta_{n}=\delta_{m}=0$, with the corresponding zero mode depicted in Figure 5a. Note that the zero mode forms a localized state, completely confined to the site of the impurity. However, the zero mode changes dramatically when the coupling between the impurity and the chain is introduced. In Figure 5b, we show the zero mode of the system under equal coupling strengths with $g_{n}=g_{m}=1$, and $\delta_{n}=\delta_{m}=0$. Here, zero mode is localized around the impurity and exhibits an exponential distribution on both sides. The spatial profile of this zero mode bears a close resemblance to the topologically protected edge state of the original SSH chain. This confirms that the impurity serves as an effective boundary for the nonreciprocal SSH chain.

In Figure 5c, we show the zero mode for the system under nonreciprocal couplings with parameters $g_{n}=g_{m}=1$, and $\delta_{n}=\delta_{m}=0.5$. It can be seen that zero mode has an asymmetric spatial distribution, with its amplitude strongly skewed to the left of the impurity site. This left-skewed localization stems directly from the nonreciprocal coupling between the impurity and SSH chain. Taken a step further, zero mode will become completely localized on the left side of the impurity when the nonreciprocal coupling strength is gradually increased to the unidirectional limit ($\delta_{n}=\delta_{m}\to 1$) as shown in Figure 5d,e. This signifies a fundamental transition in the localization of the zero mode.

Next, we introduce the other three cases of unidirectional coupling. As illustrated in Figure 5f, it is to be expected that the zero mode relocates entirely to the right side of the impurity in a unidirectional coupling regime as setting $\delta_{n}=\delta_{m}=-1$. In Figure 5g, the zero mode recovers an exponential and asymmetric decay profile centered at the impurity site as setting $\delta_{n}=-\delta_{m}=1$. It is found that its profile closely resembles that in the case of equal coupling strengths as shown in Figure 5b. In Figure 5h, the zero mode is entirely localized at the impurity site as setting $\delta_{n}=-\delta_{m}=-1$, which coincides exactly with the case in the decoupling limit as shown in Figure 5a. It follows that the zero mode is localized on the site to which the unidirectional coupling is oriented. This observation recalls studies focused on the spectrum in the previous section. Although the spectra are identical in Figure 2g and Figure 2h, the corresponding zero modes in Figure 5g and Figure 5h are strikingly different. Consequently, even if two non-Hermitian systems exhibit identical spectra, they may differ in their eigenstate structure or topological properties.

In short, it is evident that the impurity–chain coupling determines the spatial distribution of the zero mode. This has also directly evident in our analytical findings given by Equation (14) and numerical simulation as shown in Figure 5a–h. Thus, we have presented a zero mode whose spatial profile is flexibly tunable.

**Figure 6:**
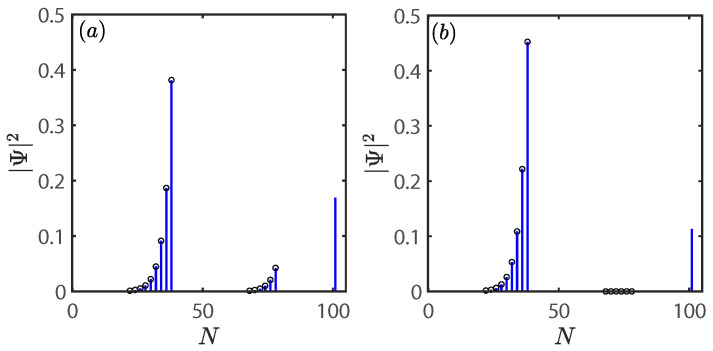
Spatial profile of zero mode for the system via A−A coupling as a function of site *N*. Nonreciprocal couplings parameters are set as $\delta_{n}=\delta_{m}=0.5$ in (**a**) and $\delta_{n}=\delta_{m}=1$ in (**b**). The results are obtained by numerically solve the Schrödinger equation. The parameters shared by all figures are taken as $L=50,n=20,m=40,t_{1}=0.2,t_{2}=1,$ and γ=0.5. The site of impurity is set to N=2L+1=101.

We now turn to briefly introduce the zero mode for the system via A−A coupling. The spatial profile of zero mode for the system (4) via A−A coupling as a function of site *N* with different impurity–chain couplings are shown in Figure 6. Nonreciprocal couplings parameters are set as $\delta_{n}=\delta_{m}=0.5$ in (a) and $\delta_{n}=\delta_{m}=1$ in (b). It can be seen that the numerical results (bars) are consistent with the analytical ones (empty circles). The corresponding analytical results for the probability amplitudes of the zero modes satisfy $A_{l}/U_{e}=0$, (see Appendix B for details)

(15) $$B_{l}/U_{e}=\left\{\begin{array}{c} 0,\;\;\;\;\;\;\;\;\;\;\;\;\;\;\;\;\;\;\;\;\;\;\;\;\;\;\;\;\;\;\;\;\;\;\;\;\;\;\;\;\;\;\;\;\;\;\;\;\;\;\;\;(l<n),\\ -Y_{4}{\left(\frac{-t_{2}}{t_{1}+\gamma }\right)}^{l-n},\;\;\;\;\;\;\;\;\;\;\;\;\;\;\;\;\;\;\;\;\;\;\;\;\;\;\;\;\;\left(n\leq l<m\right),\\ -Y_{4}{\left(\frac{-t_{2}}{t_{1}+\gamma }\right)}^{l-n}-Y_{6}{\left(\frac{-t_{2}}{t_{1}+\gamma }\right)}^{l-m},\;\;\;\;\;\;\;\;\left(m\leq l\right), \end{array}\right.$$

for $t_{1}>t_{2}+\gamma$ or $t_{1}<-t_{2}-\gamma$, and

(16) $$B_{l}/U_{e}=\left\{\begin{array}{c} Y_{4}{\left(\frac{-t_{1}-\gamma }{t_{2}}\right)}^{\left(n-l\right)}+Y_{6}{\left(\frac{-t_{1}-\gamma }{t_{2}}\right)}^{\left(m-l\right)},\;\left(l<n\right),\\ Y_{6}{\left(\frac{-t_{1}-\gamma }{t_{2}}\right)}^{\left(m-l\right)},\;\;\;\;\;\;\;\;\;\;\;\;\;\;\;\;\;\;\;\;\;\;\;\left(n\leq l<m\right),\\ 0,\;\;\;\;\;\;\;\;\;\;\;\;\;\;\;\;\;\;\;\;\;\;\;\;\;\;\;\;\;\;\;\;\;\;\;\;\;\;\;\;\;\;\;\;\;\;\;\;\;\;(m\leq l), \end{array}\right.$$

for $-t_{2}+\gamma <t_{1}<t_{2}-\gamma$. Here, $Y_{4}=\left(g_{n}+\delta_{n}\right)/\left(t_{1}+\gamma \right)$, $Y_{6}=\left(g_{m}-\delta_{m}\right)/\left(t_{1}+\gamma \right)$.

In comparison to the A−B coupling case, the spatial distribution of the zero mode is different. Note that the zero mode resides either on the left side of the impurity or between the two coupling points when the parameter satisfies $-t_{2}+\gamma <t_{1}<t_{2}-\gamma$. Conversely, it is found on the right side of the impurity or between the coupling points for $t_{1}>t_{2}+\gamma$ or $t_{1}<-t_{2}-\gamma$. Similarly, the spatial profile of zero mode can also be changed by tuning the nonreciprocity of the impurity–chain couplings. This can be clearly seen from both the analytical results given by Equation (16) and the numerical simulations as shown in Figure 6a,b. Thus, we provide a zero mode with versatile tunability in the A−A coupling case.

### 3.3. Spatial Transformation of the Bulk State

We proceed to explore the localization of the bulk states and its spatial transformation by tuning coupling parameters. The localization behavior of all bulk states can be easily quantified by the mean center of mass (mcom) of the amplitude squared of all bulk states $|\Psi_{n}\rangle$ as follows:

(17) $$\begin{array}{cc} mcom & =\frac{\sum_{\ell =1}^{N-1}\ell A\left(\ell \right)}{\sum_{\ell =1}^{N-1}A\left(\ell \right)}, \end{array}$$

with

(18) $$\begin{array}{c} A\left(\ell \right)=\frac{1}{N-1}\sum_{n=1}^{N-1}{\left|\langle \ell |\Psi_{n}\rangle \right|}^{2}. \end{array}$$

Here, we mainly focus on the distribution of bulk states living at the nonreciprocal SSH chain, so the distribution specifically on the impurity site *N* is not included in our calculations. The mcom pinpoints where the eigenstates are predominantly localized. If the mcom is precisely at the center of the chain, the eigenstates may be extended states uniformly distributed along the chain.

**Figure 7:**
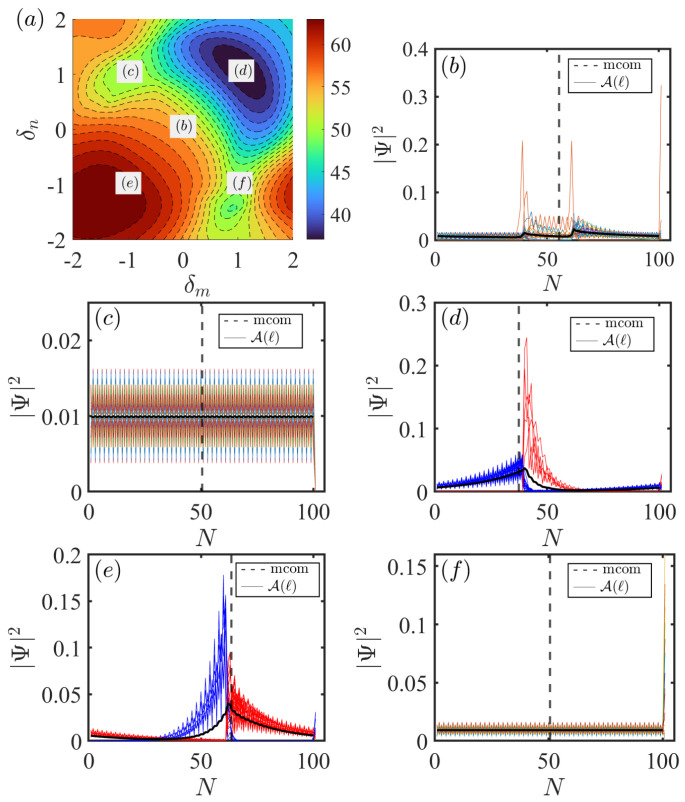
(**a**) Mcom versus the parameters $\delta_{n}$ and $\delta_{m}$ given by Equation (17) for the system via A−A coupling. (**b**–**f**) Spatial profile of all bulk states for the five points indicated in (**a**). The black dashed lines indicate the mcom, and black solid lines indicate the A(ℓ) in each case. The parameters are set as $\delta_{n}=\delta_{m}=0$ in (**b**), $\delta_{n}=-\delta_{m}=1$ in (**c**), $\delta_{n}=\delta_{m}=1$ in (**d**), $\delta_{n}=\delta_{m}=-1$ in (**e**), and $\delta_{n}=-\delta_{m}=-1$ in (**f**). The other parameters shared by all the figures are $L=50,n=20,m=31,g_{n}=1$, $g_{m}=1,t_{1}=0.2,t_{2}=1,$ and γ=0.5. The site of impurity is set to N=2L+1=101.

First, we reveal how the nonreciprocity of the impurity–chain coupling modifies the localization of the bulk states. As shown in Figure 7a, we plot the mcom given by Equation (17) as a function of impurity–chain coupling parameters $\delta_{n}$ and $\delta_{m}$ for the system (4) via A−A coupling (A−B coupling case is similar). The other parameters shared by all figures are $L=50,n=20,m=31,g_{n}=1,g_{m}=1,t_{1}=0.2,t_{2}=1,$ and γ=0.5. The site of the impurity is set to N=2L+1=101. Note that the nonreciprocal impurity–chain couplings can induce significant changes in the localization of bulk states. At first, when the nonreciprocal coupling parameters $\delta_{n}$ and $\delta_{m}$ are both negative, the bulk states primarily localize around the right coupling site N=61 (red region in Figure 7a). Upon the simultaneous reversal of the nonreciprocal directions, i.e., the sign of $\delta_{n}$ and $\delta_{m}$ flip from negative to positive, the bulk states gradually shifts from the right to the left coupling site N=39 (blue region in Figure 7a). Conversely, the bulk states become more delocalized in other regions of the parameter space (green region in Figure 7a).

In Figure 7b–f, we show the spatial profile of all bulk states for the five typical points indicated in Figure 7a.The black dashed lines denote the mcom, and black solid lines denote the A(ℓ) in each case. Figure 7b shows that when the nonreciprocity of impurity–chain couplings vanishes ($\delta_{n(m)}=0$), a few eigenstates appear as isolated, highly localized peaks, while the majority display extended distributions with exponential decay along the chain. More interestingly, increasing the nonreciprocity causes the system to exhibit a different localization behavior. In Figure 7c, we show all bulk states in unidirectional coupling limits with opposite directions ($\delta_{n}=-\delta_{m}=1$). It results in a delocalized, uniform distribution of eigenstates along the nonreciprocal SSH chain when two coupling directions all pointing toward the chain. Note that all eigenstates have exactly disappearing amplitudes on the impurity site. This corresponds to a mcom ≈50 in Figure 7a (green region). Additionally, Figure 7f shows a comparable case where both coupling directions point toward the impurity ($\delta_{n}=-\delta_{m}=-1$). It can be observed that the bulk states are uniformly distributed along the chain but form a prominent peak at the impurity site.

In Figure 7d, we show all bulk states in the unidirectional coupling regime with same directions ($\delta_{n}=\delta_{m}=1$). In this case, both coupling directions are oriented toward the left coupling site. This corresponds to a mcom ≈39 in Figure 7a. As a result, all bulk states are localized around the left coupling site N=39. Unexpectedly, the bulk states are divided into two distinct classes by the left coupling site, with one group mainly on the left (indicated by blue lines) and the other on the right (indicated by red lines). Another feature is that the two types of bulk states differ in their degree of localization, with those on the right being more strongly localized. This implies that the system supports two bulk-state localization regimes, characterized by the distinct spatial distribution and the degree of localization. In Figure 7e, upon changing coupling directions by setting $\delta_{n}=\delta_{m}=-1$, the localization is rapidly inverted and all bulk states pile up towards the right coupling site N=61. This corresponds to a mcom ≈61 in Figure 7a. Again, two distinct types of bulk states are found localized near the right coupling site.

The unusual localization behavior originates from the non-Hermiticity of the system. The nonreciprocal coupling in the SSH chain serves as the fundamental condition for the emergence of localized bulk states. Moreover, the impurity–chain unidirectional coupling creates two distinct boundaries, which guarantees that the bulk states become localized near one boundary. Two boundaries also effectively force the original chain to split into two independent chains as shown in Figure 1. These provide the condition for the emergence of two distinct types of localized bulk states. Hence, the nonreciprocal SSH chain and the unidirectional coupling to the impurity jointly leads to forming two types of localized modes at the same boundary.

In short, the system can be controlled by adjusting the nonreciprocity of impurity–chain couplings. Particularly, it exhibits striking localization behaviors in unidirectional coupling limits.

Next, we examine how the impurity–chain coupling strength impacts the localization of bulk states. Meanwhile, the system is slightly adjusted for simplicity. We extend the nonreciprocal SSH chain by introducing nonreciprocity to its intercell couplings, with the corresponding Hamiltonian given by

(19) $$\begin{array}{c} H_{\mathrm{SSH}}^{\prime }=\sum_{l=1}^{L}\left[\left(t_{1}+\gamma \right){\hat{C}}_{A,l}^{†}{\hat{C}}_{B,l}+\left(t_{1}-\gamma \right){\hat{C}}_{B,l}^{†}{\hat{C}}_{A,l}+\left(t_{2}+\gamma \right){\hat{C}}_{B,l}^{†}{\hat{C}}_{A,l+1}\left(t_{2}-\gamma \right){\hat{C}}_{A,l+1}^{†}{\hat{C}}_{B,l}\right]. \end{array}$$

This facilitates the uniform tuning of the nearest-neighbor couplings in the nonreciprocal SSH chain. With Equation (2), the impurity–chain Hamiltonian via A−B couplings can be rewritten as

(20) $$H_{AB}^{\prime }=H_{\mathrm{SSH}}^{\prime }+H_{I,AB},$$

with

(21) $$\begin{array}{c} H_{I,AB}=\left(g_{1}-\delta_{1}\right)\sigma^{+}{\hat{C}}_{A,1}+\left(g_{1}+\delta_{1}\right)\sigma^{-}{\hat{C}}_{A,1}^{†}+\left(g_{L}+\delta_{L}\right)\sigma^{+}{\hat{C}}_{B,L}+\left(g_{L}-\delta_{L}\right)\sigma^{-}{\hat{C}}_{B,L}^{†}. \end{array}$$

At this stage, the nonreciprocity extends to every couplings in the system. The impurity–chain coupling sites are also pinned to the two joined ends of the chain with n=1, m=L. This ensures that the rest of the chain remains intact and is not split into two separate parts.

**Figure 8:**
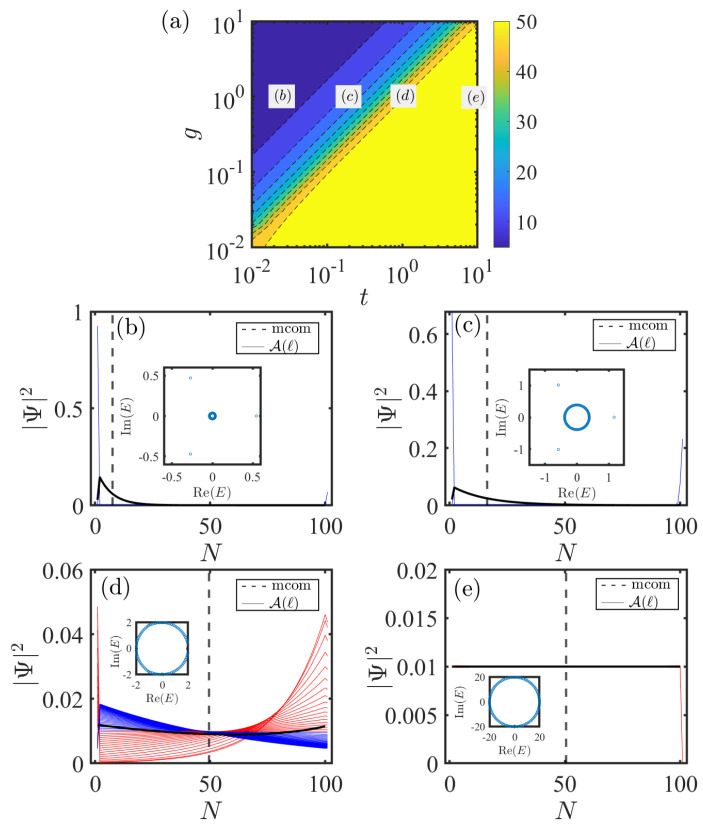
(**a**) Mcom versus the parameters *g* and *t* given by Equation (17) for the system (Equation (20)) in the unidirectional coupling regime. (**b**–**e**) Spatial profile of all bulk states for the four points indicated in (**a**). The black dashed lines indicate the mcom, and black solid lines indicate the A(ℓ) in each case. The parameters are set as t=0.02, g=1 in (**b**), t=0.2, g=1 in (**c**), t=1, g=1 in (**d**), and t=10, g=1 in (**e**). The parameters shared by all the figures are L=50,n=1,m=50, $g_{n}=g_{m}=g,\delta_{n}=\delta_{m}=g,t_{1}=t_{2}=t,$ and γ=t. The site of impurity is set to N=2L+1=101.

As shown in Figure 8a, we plot the mcom on the parameter space of *g* and *t* for the system (Equation (20)) via A−B coupling. In order to substantially increase the non-Hermiticity of systems, we set all nonreciprocal couplings to the unidirectional limit. Hence, the parameters are taken as $g_{1}=\delta_{1}=g,g_{L}=\delta_{L}=g$, and $t_{1}=t_{2}=\gamma =t$. Here, the parameter 2g characterizes the unidirectional impurity–chain coupling strength, and 2t describes the unidirectional nearest-neighbor coupling strength in the chain. A key observation from the Figure 8a is that the mcom is driven to smaller values as increasing impurity–chain coupling strengths and decreasing intra-chain coupling strengths. For example, mcom≈10 for g=10 and t=0.01. This implies that the bulk states are mainly localized around the site N=10. In contrast, the mcom grows progressively to about 50 as the impurity–chain couplings decrease and the intra-chain couplings increase. This suggests a progressive weakening in the localization of the bulk states. For example, mcom≈50 for g=0.01 and t=10. In this case, the mcom is precisely at the center of the chain. It means that the bulk states may be extended states uniformly distributed along the chain. Therefore, the couplings between the impurity and the chain provides an effective boundary condition for the nonreciprocal SSH chain. The localization of bulk states is enhanced under strong impurity–chain couplings and weak intra-chain couplings.

We now illustrate the features of the bulk states through a few specific cases. In Figure 8b–e, we show the spatial profile of all bulk states for the four typical points indicated in (a). The black dashed lines denote the mcom, and black solid lines denote the A(ℓ) in each case. Figure 8b–e show that the localization of bulk states is tunable by changing the impurity–chain couplings and intra-chain couplings, in full agreement with the above discussion. Interestingly, the transition in the localization of bulk states is non-trivial. As shown in Figure 8b, most bulk states accumulate at the left end of the chain together when t=0.02, g=1 (coincide with the black solid line). Furthermore, a minority of states are strongly localized near the impurity (blue solid line). Remarkably, in addition to the typical non-Hermitian skin effect, it gives rise to a class of bound states induced by the impurity. The corresponding spectrum is provided in the inset of the Figure 8b. Note that there are three isolated eigenvalues outside the continuous energy bands. These correspond to the bound states induced by the impurity. Next, Figure 8c shows that the bulk states become progressively less localized as the intra-chain couplings increase (t=0.2, g=1). Notably, the bulk states exhibit a bipolar localization when the intra-chain and impurity–chain coupling strengths become comparable (t=g=1) as shown in Figure 8d. Some of the bulk states are localized at the left end of the chain (blue line), while others are localized at the right end (red line). In addition, the bound states localized near the impurity vanish in this case, along with their corresponding isolated eigenvalues outside the energy band. Finally, the bulk states transform into extended states uniformly distributed along the chain once the intra-chain couplings become significantly larger than the impurity–chain couplings (t=10,g=1) as shown in Figure 8e.

In short, the bulk states witness a remarkable transformation in their localization as the coupling strength varies, characterized by a transition from conventional skin states to bipolar skin states. Generally, this distinct localization behaviors of bulk states in non-Hermitian topological systems can be distinguished by the generalized Brillouin zone [16]. Next, we consider the real-space eigenequation of the system in the bulk of the chain, which satisfies

(22) $$\begin{array}{c} \left(t_{2}-\gamma \right)\psi_{B,l-1}+\left(t_{1}+\gamma \right)\psi_{B,l}=E\psi_{A,l},\\ \left(t_{1}-\gamma \right)\psi_{A,l}+\left(t_{2}+\gamma \right)\psi_{A,l+1}=E\psi_{B,l}. \end{array}$$

**Figure 9:**
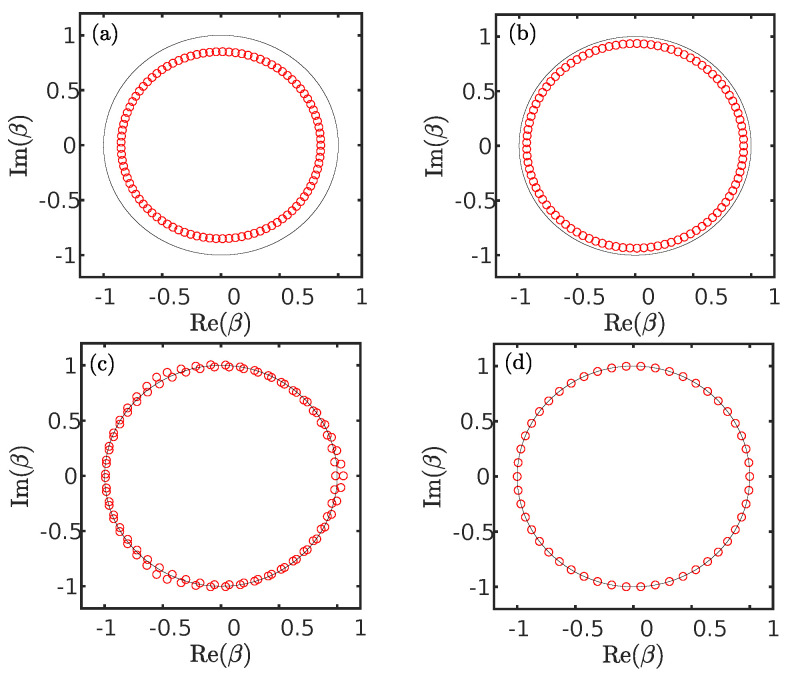
$\beta_{1}$ (red circle) given by Equation (23) for the system (Equation (20)) in the unidirectional coupling regime. The black circle denotes the unit circle. The parameters in (**a**–**d**) are identical to those in Figure 8b–e.

With $\left(\psi_{A,l},\psi_{B,l}\right)=\beta^{l}\left(\psi_{A},\psi_{B}\right)$, it yields

(23) $$\beta_{1,2}\left(E\right)=\frac{\left[\Delta \pm \sqrt{\Delta^{2}-4\left(t_{2}^{2}-\gamma^{2}\right)\left(t_{1}^{2}-\gamma^{2}\right)}\right]}{2\left(t_{2}+\gamma \right)\left(t_{1}+\gamma \right)},$$

where $\Delta =E^{2}+2\gamma^{2}-t_{1}^{2}-t_{2}^{2}$, and +(−) corresponds to $\beta_{1}\left(\beta_{2}\right)$. Here, we adopt the generalized Bloch phase factor β, whose modulus encodes the spatial decay of the wave function, as a replacement for the conventional Bloch factor $e^{ik}$. In Figure 9a–d, we show $\beta_{1}$ (red circle) for the system (20) in the unidirectional coupling regime. The parameters are identical to those in Figure 8b–e. For the system under periodic boundary conditions without the impurity, $|\beta_{1}|=1$ corresponds to the black unit circle in Figure 9a–d, and the wave functions take the form of skin-free states. As shown in Figure 9a,b, the Bloch fact $\beta_{1}$ lies inside the unit circle. It implies that the corresponding bulk states are localized at the left end of the chain. Interestingly, the values of $\beta_{1}$ are partially distributed inside and partially outside the unit circle as shown in Figure 9c, which suggests that bulk states are localized at the left ($|\beta_{1}|<1$) or the right ($|\beta_{1}|>1$) end of the chain. Eventually, $\beta_{1}$ becomes nearly coincident with the unit circle in Figure 9d. In this case, the bulk states obviously take the form of skin-free states. Thus, generalized Bloch phase factor β serves as an effective probe for the localization behavior of bulk states.

## 4. Discussion and Conclusions

In summary, we have studied an impurity coupled to two sites of a nonreciprocal SSH chain. The key feature is that the impurity–chain coupling is non-Hermitian. We present the spectral structure of the system and provide the conditions for the emergence of zero modes under various coupling regimes. Remarkably, the unidirectional impurity–chain coupling serves as a mechanism for inducing a shift in the parameter regime for the zero mode. The localization of the zero mode is also highly tunable, controlled by changing the nonreciprocity of the coupling between the impurity and the chain. We further characterize the spatial distribution of bulk states via the mcom. A striking finding is that two distinct types of bulk states coexist at the same boundary in the unidirectional coupling regime. We also show that the bulk states undergo a remarkable transformation in their localization as the coupling strength varies, marked by a transition from conventional skin states to bipolar skin states. This study provides a theoretical framework for controlling non-Hermitian topological systems via coupling impurities, revealing novel phenomena in nonreciprocal models.

We propose an experimental scheme based on electrical circuits. Crucially, nonreciprocal couplings between the impurity and the chain are engineered by placing an effective nonreciprocal capacitance ($C_{i}\pm C_{j}$). The capacitance can vary over a broad range (e.g., from pF to nF), making the transition of nonreciprocal coupling readily achievable in the experiment.

## Figures and Tables

**Table 1:** A table with four different conditions for the emergence of zero energy.

	Condition	Parameter Range	Figure	*v*
I	α≠0, β≠0	$-t_{2}+\gamma <t_{1}<t_{2}-\gamma$	Figure 2b–d	1
II	α=0, β≠0	$-t_{2}-\gamma <t_{1}<t_{2}-\gamma$	Figure 2e	$\frac{1}{2}$, 1
III	α≠0, β=0	$-t_{2}+\gamma <t_{1}<t_{2}+\gamma$	Figure 2f	$\frac{1}{2}$, 1
IV	α=0, β=0	$t_{1}\in$ arbitrary value	Figure 2g,h	0, $\frac{1}{2}$, 1

## Data Availability

The data are contained within the article.

## Appendix A. Energy Equation

We consider a system where an impurity coupled to a nonreciprocal SSH chain via A−B coupling. In the single-excitation subspace, the eigenstate of the Bloch Hamiltonian in momentum space can be written as

(A1) $$\begin{array}{c} |\psi \rangle =U_{e}|e,G\rangle +\sum_{k}A_{k}{\hat{C}}_{A,k}^{†}|g,G\rangle +\sum_{k}{B_{k}}^{†}{\hat{C}}_{B,k}|g,G\rangle , \end{array}$$

where |G〉 denotes the ground state of the SSH chain. $A_{k}\left(B_{k}\right)$ denotes the amplitude in site A(B) of the *k*th unit cell, and $U_{e}$ denotes the amplitude in site of the impurity. The time-independent Schrödinger equation $H_{AB}\left(k\right)|\psi \rangle =E\left(k\right)|\psi \rangle$ together with Equation (A1) leads to,

(A2) $$\begin{array}{cc} \; & EU_{e}=\frac{1}{\sqrt{L}}\sum_{k}\left[A_{k}\left(g_{n}-\delta_{n}\right)e^{ikn}+B_{k}\left(g_{m}+\delta_{m}\right)e^{ikm}\right],\\ \; & EA_{k}=\left(t_{1}+\gamma +t_{2}e^{-ik}\right)B_{k}+\frac{g_{n}+\delta_{n}}{\sqrt{L}}e^{-ikn}U_{e},\\ \; & EB_{k}=\left(t_{1}-\gamma +t_{2}e^{ik}\right)A_{k}+\frac{g_{m}-\delta_{m}}{\sqrt{L}}e^{-ikm}U_{e}. \end{array}$$

Eliminating $A_{k}$,$B_{k}$ and $U_{e}$ in Equation (A2), we obtain the equation for *E*,

(A3) $$\begin{array}{cc} E & =\frac{1}{L}\sum_{k}\frac{\left(g_{n}-\delta_{n}\right)e^{ikn}}{E^{2}-\omega_{k}^{2}}\left[\left(g_{n}+\delta_{n}\right)e^{-ikn}E+\left(g_{m}-\delta_{m}\right)e^{-ikm}\left(t_{1}+\gamma +t_{2}e^{-ik}\right)\right]\\ \; & +\frac{1}{L}\sum_{k}\frac{\left(g_{m}+\delta_{m}\right)e^{ikm}}{E^{2}-\omega_{k}^{2}}\left[\left(g_{m}-\delta_{m}\right)e^{-ikm}E+\left(g_{n}+\delta_{n}\right)e^{-ikn}\left(t_{1}-\gamma +t_{2}e^{ik}\right)\right].\\ \; & =\frac{1}{2\pi }\int_{-\pi }^{\pi }dk\frac{\left(g_{n}-\delta_{n}\right)e^{ikn}}{E^{2}-\omega_{k}^{2}}\left[\left(g_{n}+\delta_{n}\right)e^{-ikn}E+\left(g_{m}-\delta_{m}\right)e^{-ikm}\left(t_{1}+\gamma +t_{2}e^{-ik}\right)\right]\\ \; & +\frac{1}{2\pi }\int_{-\pi }^{\pi }dk\frac{\left(g_{m}+\delta_{m}\right)e^{ikm}}{E^{2}-\omega_{k}^{2}}\left[\left(g_{m}-\delta_{m}\right)e^{-ikm}E+\left(g_{n}+\delta_{n}\right)e^{-ikn}\left(t_{1}-\gamma +t_{2}e^{ik}\right)\right]. \end{array}$$

To obtain the zero mode, we set E=0 and write $z_{1}=e^{ik}$, $z_{2}=e^{-ik}$. It yields

(A4) $$\begin{array}{cc} 0= & \frac{-\left(g_{m}+\delta_{m}\right)\left(g_{n}+\delta_{n}\right)}{2\pi i}\left[\oint_{|z_{1}|=1}dz_{1}\frac{\left(t_{1}-\gamma \right)z_{1}^{m-n}+t_{2}z_{1}^{m-n+1}}{\left(t_{1}+\gamma \right)t_{2}\left(z_{1}+\frac{t_{2}}{t_{1}+\gamma }\right)\left(z_{1}+\frac{t_{1}-\gamma }{t_{2}}\right)}\right]\\ \; & +\frac{-\left(g_{m}-\delta_{m}\right)\left(g_{n}-\delta_{n}\right)}{2\pi i}\left[\oint_{|z_{2}|=1}dz_{2}\frac{\left(t_{1}+\gamma \right)z_{2}^{m-n}+t_{2}z_{2}^{m-n+1}}{\left(t_{1}-\gamma \right)t_{2}\left(z_{2}+\frac{t_{2}}{t_{1}-\gamma }\right)\left(z_{2}+\frac{t_{1}+\gamma }{t_{2}}\right)}\right]. \end{array}$$

Then, Equation (A4) can be simplified via the residue theorem as

(A5) $$\begin{array}{cc} 0= & \left\{\begin{array}{cc} \frac{\alpha }{t_{2}}{\left(\frac{-t_{2}}{t_{1}-\gamma }\right)}^{m-n+1}, & (-t_{2}-\gamma <t_{1}<-t_{2}+\gamma ),\\ 0, & (-t_{2}+\gamma <t_{1}<t_{2}-\gamma ),\\ \frac{\beta }{t_{2}}{\left(\frac{-t_{2}}{t_{1}+\gamma }\right)}^{m-n+1}, & (t_{2}-\gamma <t_{1}<t_{2}+\gamma ),\\ \frac{\alpha }{t_{2}}{\left(\frac{-t_{2}}{t_{1}-\gamma }\right)}^{m-n+1}+\frac{\beta }{t_{2}}{\left(\frac{-t_{2}}{t_{1}+\gamma }\right)}^{m-n+1}, & (t_{1}<-t_{2}-\gamma \;or\;t_{1}>t_{2}+\gamma ). \end{array}\right. \end{array}$$

Here, $\alpha =\left(g_{m}-\delta_{m}\right)\left(g_{n}-\delta_{n}\right)$, $\beta =\left(g_{m}+\delta_{m}\right)\left(g_{n}+\delta_{n}\right)$, and it is assumed that $t_{2}>\gamma >0$.

For the system via A−A coupling, the interaction Hamiltonian in the momentum space reads

(A6) $$\begin{array}{c} H_{AA}\left(k\right)=H_{\mathrm{SSH}}\left(k\right)+H_{I,AA}\left(k\right), \end{array}$$

with

(A7) $$\begin{array}{cc} H_{I,AA}\left(k\right)=\frac{1}{\sqrt{L}}\sum_{k} & [\left(g_{n}-\delta_{n}\right)e^{ikn}\sigma^{+}{\hat{C}}_{A,k}+\left(g_{n}+\delta_{n}\right)e^{-ikn}\sigma^{-}{\hat{C}}_{A,k}^{†}\\ \; & +\left(g_{m}+\delta_{m}\right)e^{ikm}\sigma^{+}{\hat{C}}_{A,k}+\left(g_{m}-\delta_{m}\right)e^{-ikm}\sigma^{-}{\hat{C}}_{A,k}^{†}]. \end{array}$$

With $H_{AA}\left(k\right)|\psi \rangle =E\left(k\right)|\psi \rangle$, we have

(A8) $$\begin{array}{cc} EU_{e}= & \frac{1}{\sqrt{L}}\sum_{k}A_{k}\left[\left(g_{n}-\delta_{n}\right)e^{ikn}+\left(g_{m}+\delta_{m}\right)e^{ikm}\right],\\ EA_{k}= & \frac{1}{\sqrt{L}}\left[\left(g_{n}+\delta_{n}\right)e^{-ikn}+\left(g_{m}-\delta_{m}\right)e^{-ikm}\right]U_{e}+\left(t_{1}+\gamma +t_{2}e^{-ik}\right)B_{k},\\ EB_{k}= & \left(t_{1}-\gamma +t_{2}e^{ik}\right)A_{k}. \end{array}$$

Some algebras shows that the energy *E* satisfies

(A9) $$\begin{array}{cc} E=\frac{1}{L}\sum_{k} & \frac{\left(g_{n}-\delta_{n}\right)e^{ikn}}{E^{2}-\omega_{k}^{2}}\left[\left(g_{n}+\delta_{n}\right)e^{-ikn}E+\left(g_{m}-\delta_{m}\right)e^{-ikm}\left(t_{1}+\gamma +t_{2}e^{-ik}\right)\right]\\ +\frac{1}{L}\sum_{k} & \frac{\left(g_{m}+\delta_{m}\right)e^{ikm}}{E^{2}-\omega_{k}^{2}}\left[\left(g_{m}-\delta_{m}\right)e^{-ikm}E+\left(g_{n}+\delta_{n}\right)e^{-ikn}\left(t_{1}-\gamma +t_{2}e^{ik}\right)\right]. \end{array}$$

Evidently, E=0 is always the solution of the Equation (A9).

## Appendix B. Analytical Results of Zero Modes

For the system via A−B couplings, from the Equation (A2), the probability amplitudes $A_{k}$ and $B_{k}$ satisfy

(A10) $$\begin{array}{cc} A_{k}/U_{e}=\frac{f(k)}{\sqrt{L}t_{2}} & [\left(t_{1}+\gamma +t_{2}e^{-ik}\right)\left(g_{m}-\delta_{m}\right)e^{-ikm}+E\left(g_{n}+\delta_{n}\right)e^{-ikn}],\\ B_{k}/U_{e}=\frac{f(k)}{\sqrt{L}t_{2}} & [\left(t_{1}-\delta +t_{2}e^{ik}\right)\left(g_{n}+\delta_{n}\right)e^{-ikn}+E\left(g_{m}-\delta_{m}\right)e^{-ikm}], \end{array}$$

where $f\left(k\right)=1/(x-\left(t_{1}+\gamma \right)e^{ik}-\left(t_{1}-\gamma \right)e^{-ik})$ and $x=\left(E^{2}-\left(t_{1}+\gamma \right)\left(t_{1}-\gamma \right)-t_{2}^{2}\right)/t_{2}$. By Fourier expansion for f(k),

(A11) $$f\left(k\right)=a_{0}+\sum_{p}\left[\left(\frac{a_{p}-ib_{p}}{2}\right)e^{ikp}+\left(\frac{a_{p}+ib_{p}}{2}\right)e^{-ikp}\right],$$

with

(A12) $$\begin{array}{cc} a_{0} & =\frac{1}{2\pi }\int_{-\pi }^{\pi }f\left(k\right)dk,\\ \frac{a_{p}+ib_{p}}{2} & =\frac{1}{2\pi }\int_{-\pi }^{\pi }f\left(k\right)e^{ikp}dk,\\ \frac{a_{p}-ib_{p}}{2} & =\frac{1}{2\pi }\int_{-\pi }^{\pi }f\left(k\right)e^{-ikp}dk, \end{array}$$

we derive f(k) as follows

(A13) $$f\left(k\right)=\frac{{\left(-1\right)}^{y+1}\left[1+\sum_{p=1}^{L}\left(e^{-ikp}a^{p}+e^{ikp}b^{p}\right)\right]}{\sqrt{x^{2}-4\left(t_{1}+\gamma \right)\left(t_{1}-\gamma \right)}},$$

where y=θ(x) is the step function, and $a=\left(x+\sqrt{x^{2}-4\left(t_{1}+\gamma \right)\left(t_{1}-\gamma \right)}\right)/2\left(t_{1}+\gamma \right)$, $b=\left(x+\sqrt{x^{2}-4\left(t_{1}+\gamma \right)\left(t_{1}-\gamma \right)}\right)/2\left(t_{1}-\gamma \right)$ for $x<-2|t_{1}|$ or $a=\left(x-\sqrt{x^{2}-4\left(t_{1}+\gamma \right)\left(t_{1}-\gamma \right)}\right)/2\left(t_{1}+\gamma \right),b=\left(x-\sqrt{x^{2}-4\left(t_{1}+\gamma \right)\left(t_{1}-\gamma \right)}\right)/2\left(t_{1}-\gamma \right)$ for $x>2|t_{1}|$. Substituting the above results into Equation (A10), we will get the probability amplitude in real space by inverse Fourier transformation. The result is,

(A14) $$\begin{array}{cc} \; & A_{l}/U_{e}=\frac{1}{\sqrt{L}}\sum_{k}e^{ikl}A_{k}/U_{e}\\ \; & =\frac{{\left(-1\right)}^{y+1}\left(T_{n}\tau_{1}^{\left|l-n\right|}+\left(g_{m}-\delta_{m}\right)\tau_{2}^{\left|l-m-1\right|}+Y_{2}\tau_{2}^{\left|l-m\right|}\right)}{\sqrt{x^{2}-4\left(t_{1}+\gamma \right)\left(t_{2}-\gamma \right)}}, \end{array}$$

(A15) $$\begin{array}{cc} \; & B_{l}/U_{e}=\frac{1}{\sqrt{L}}\sum_{k}e^{ikl}B_{k}/U_{e}\\ \; & =\frac{{\left(-1\right)}^{y+1}\left(T_{m}\tau_{2}^{\left|l-m\right|}+\left(g_{n}+\delta_{n}\right)\tau_{1}^{\left|l-n+1\right|}+Y_{1}\tau_{1}^{\left|l-n\right|}\right)}{\sqrt{x^{2}-4\left(t_{1}+\gamma \right)\left(t_{1}-\gamma \right)}}, \end{array}$$

where $T_{n}=\left(g_{n}+\delta_{n}\right)E/t_{2}$, $T_{m}=\left(g_{m}-\delta_{m}\right)E/t_{2}$, $Y_{1}=\left(g_{n}+\delta_{n}\right)\left(t_{1}-\gamma \right)/t_{2}$, $Y_{2}=\left(g_{m}-\delta_{m}\right)\left(t_{1}+\gamma \right)/t_{2}$. $\tau_{1}=a$ for l≥n, $\tau_{1}=b$ for l<n. $\tau_{2}=a$ for l>m, $\tau_{2}=b$ for l≤m. For the zero modes with E=0, we would have $x<-2|t_{1}|$, thus *a* and *b* are reduced to $-\left(t_{1}-\gamma \right)/t_{2}$ and $-\left(t_{1}+\gamma \right)/t_{2}$ for $-t_{2}+\gamma <t_{1}<t_{2}-\gamma$, respectively. *a* and *b* are reduced to $-t_{2}/\left(t_{1}+\gamma \right)$ and $-t_{2}/\left(t_{1}-\gamma \right)$ for $t_{1}>t_{2}+\gamma$ or $t_{1}<-t_{2}-\gamma$, respectively. Then the Equations (A14) and (A15) can be simplified as

(A16) $$\begin{array}{cc} \; & A_{l}/U_{e}=Y_{3}\times \left\{\begin{array}{cc} {\left(-\frac{t_{1}-\gamma }{t_{2}}\right)}^{\left(l-m\right)}, & (l>m),\\ 0, & (l\leq m), \end{array}\right. \end{array}$$

(A17) $$\begin{array}{cc} \; & B_{l}/U_{e}=Y_{4}\times \left\{\begin{array}{cc} {\left(-\frac{t_{1}+\gamma }{t_{2}}\right)}^{\left(n-l\right)}, & (l<n),\\ 0, & (l\geq n), \end{array}\right. \end{array}$$

where $Y_{3}=\left(g_{m}-\delta_{m}\right)/\left(t_{1}-\gamma \right)$, $Y_{4}=\left(g_{n}+\delta_{n}\right)/\left(t_{1}+\gamma \right)$.

Similarly, for the system via A−A coupling, Equation (A8) yields

(A18) $$\begin{array}{cc} A_{k}/U_{e}= & \frac{f(k)E}{\sqrt{L}t_{2}}\left[\left(g_{n}+\delta_{n}\right)e^{-ikn}+\left(g_{m}-\delta_{m}\right)e^{-ikm}\right],\\ B_{k}/U_{e}= & \frac{f\left(k\right)\left(t_{1}-\gamma +t_{2}e^{ik}\right)}{\sqrt{L}t_{2}}\left[\left(g_{n}+\delta_{n}\right)e^{-ikn}+\left(g_{m}-\delta_{m}\right)e^{-ikm}\right]. \end{array}$$

By the inverse Fourier transformation, we can obtain

(A19) $$\begin{array}{cc} A_{l}/U_{e} & =\frac{1}{\sqrt{L}}\sum_{k}e^{ikl}A_{k}/U_{e}\\ \; & =\frac{{\left(-1\right)}^{y+1}\left(T_{n}\tau_{1}^{\left|l-n\right|}+T_{m}\tau_{3}^{\left|l-m\right|}\right)}{\sqrt{x^{2}-4\left(t_{1}+\gamma \right)\left(t_{1}-\gamma \right)}}, \end{array}$$

(A20) $$\begin{array}{cc} B_{l}/U_{e} & =\frac{1}{\sqrt{L}}\sum_{k}e^{ikl}B_{k}/U_{e}\\ \; & =\frac{{\left(-1\right)}^{y+1}}{\sqrt{x^{2}-4\left(t_{1}+\gamma \right)\left(t_{1}-\gamma \right)}}\left\{Y_{1}\tau_{1}^{\left|l-n\right|}+Y_{5}\tau_{3}^{\left|l-m\right|}\right.\\ \; & \left.+\left(g_{n}+\delta_{n}\right)\tau_{1}^{\left|l-n+1\right|}+\left(g_{m}-\delta_{m}\right)\tau_{3}^{\left|l-m+1\right|}\right\}, \end{array}$$

where $Y_{5}=\left(g_{m}-\delta_{m}\right)\left(t_{1}-\gamma \right)/t_{2}$, $\tau_{3}=a$ for l≥m, $\tau_{3}=b$ for l<m. Then setting E=0, the Equations (A19) and (A20) can be simplified as $A_{l}/U_{e}=0$,

(A21) $$B_{l}/U_{e}=\left\{\begin{array}{c} 0,\;\;\;\;\;\;\;\;\;\;\;\;\;\;\;\;\;\;\;\;\;\;\;\;\;\;\;\;\;\;\;\;\;\;\;\;\;\;\;\;\;\;\;\;\;\;\;\;\;\;\;\;\;\;\;\;\;(l<n),\\ -Y_{4}{\left(\frac{-t_{2}}{t_{1}+\gamma }\right)}^{l-n},\;\;\;\;\;\;\;\;\;\;\;\;\;\;\;\;\;\;\;\;\;\;\;\;\;\;\;\;\;\;\;\left(n\leq l<m\right),\\ -Y_{4}{\left(\frac{-t_{2}}{t_{1}+\gamma }\right)}^{l-n}-Y_{6}{\left(\frac{-t_{2}}{t_{1}+\gamma }\right)}^{l-m},\;\;\;\;\;\;\;\;\;\;\;\;\;\;\left(m\leq l\right), \end{array}\right.$$

for $t_{1}>t_{2}+\gamma$ or $t_{1}<-t_{2}-\gamma$, and

(A22) $$B_{l}/U_{e}=\left\{\begin{array}{c} Y_{4}{\left(\frac{-t_{1}-\gamma }{t_{2}}\right)}^{\left(n-l\right)}+Y_{6}{\left(\frac{-t_{1}-\gamma }{t_{2}}\right)}^{\left(m-l\right)},\;\left(l<n\right),\\ Y_{6}{\left(\frac{-t_{1}-\gamma }{t_{2}}\right)}^{\left(m-l\right)},\;\;\;\;\;\;\;\;\;\;\;\;\;\;\;\;\;\;\;\;\;\left(n\leq l<m\right),\\ 0,\;\;\;\;\;\;\;\;\;\;\;\;\;\;\;\;\;\;\;\;\;\;\;\;\;\;\;\;\;\;\;\;\;\;\;\;\;\;\;\;\;\;\;\;\;\;\;\;(m\leq l), \end{array}\right.$$

for $-t_{2}+\gamma <t_{1}<t_{2}-\gamma$. Here, $Y_{6}=\left(g_{m}-\delta_{m}\right)/\left(t_{1}+\gamma \right)$.
